# *N*′-(5-Chloro-2-hy­droxy­benzyl­idene)-2-meth­oxy­benzohydrazide

**DOI:** 10.1107/S160053681002180X

**Published:** 2010-06-16

**Authors:** Yu-Mei Hao

**Affiliations:** a Department of Chemistry, Baicheng Normal University, Baicheng 137000, People’s Republic of China

## Abstract

The title Schiff base compound, C_15_H_13_ClN_2_O_3_, was prepared by the reaction of equimolar quanti­ties of 5-chloro-2-hy­droxy­benzaldehyde with 2-meth­oxy­benzohydrazide in a methanol solution. The dihedral angle between the two benzene rings is 20.6 (3)°. An intra­molecular O—H⋯N hydrogen bond may influence the mol­ecular conformation. In the crystal structure, mol­ecules form chains along the *b* direction *via* inter­molecular N—H⋯O hydrogen bonds which are bifurcated involving an intra­molecular N—H⋯O hydrogen bond.

## Related literature

For the pharmaceutical and medicinal activities of Schiff bases, see: Sriram *et al.* (2006 ▶); Karthikeyan *et al.* (2006 ▶); Dao *et al.* (2000 ▶). For the coordination chemistry of Schiff bases, see: Ali *et al.* (2008 ▶); Kargar *et al.* (2009 ▶); Yeap *et al.* (2009 ▶). For the crystal structures of Schiff base compounds, see: Fun *et al.* (2009 ▶); Nadeem *et al.* (2009 ▶); Eltayeb *et al.* (2008 ▶). For the structures of related Schiff base compounds previously reported by the author, see: Hao (2009*a* ▶,*b* ▶,*c* ▶,**d* ▶, *2010 ▶). For standard bond-length data, see: Allen *et al.* (1987 ▶).
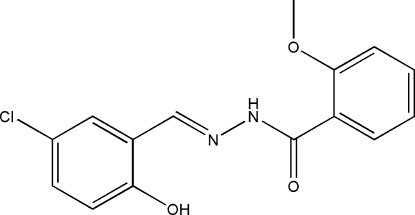


## Experimental

### 

#### Crystal data

C_15_H_13_ClN_2_O_3_
*M* *_r_* = 304.72
Orthorhombic, 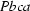

*a* = 15.392 (3) Å
*b* = 9.110 (2) Å
*c* = 20.128 (3) Å
*V* = 2822.4 (9) Å^3^
*Z* = 8
Mo *K*α radiation
μ = 0.28 mm^−1^
*T* = 298 K
0.30 × 0.30 × 0.27 mm

#### Data collection

Bruker SMART CCD area-detector diffractometer
Absorption correction: multi-scan (*SADABS*; Sheldrick, 1996 ▶) *T*_min_ = 0.920, *T*_max_ = 0.928
9958 measured reflections
3051 independent reflections
1463 reflections with *I* > 2σ(*I*)
*R*_int_ = 0.067

#### Refinement

*R*[*F* ^2^ > 2σ(*F* ^2^)] = 0.056
*wR*(*F* ^2^) = 0.150
*S* = 0.99
3051 reflections
195 parameters
1 restraint
H atoms treated by a mixture of independent and constrained refinement
Δρ_max_ = 0.17 e Å^−3^
Δρ_min_ = −0.27 e Å^−3^

### 

Data collection: *SMART* (Bruker, 2002 ▶); cell refinement: *SAINT* (Bruker, 2002 ▶); data reduction: *SAINT*; program(s) used to solve structure: *SHELXS97* (Sheldrick, 2008 ▶); program(s) used to refine structure: *SHELXL97* (Sheldrick, 2008 ▶); molecular graphics: *SHELXTL* (Sheldrick, 2008 ▶); software used to prepare material for publication: *SHELXL97*.

## Supplementary Material

Crystal structure: contains datablocks global, I. DOI: 10.1107/S160053681002180X/lh5065sup1.cif

Structure factors: contains datablocks I. DOI: 10.1107/S160053681002180X/lh5065Isup2.hkl

Additional supplementary materials:  crystallographic information; 3D view; checkCIF report

## Figures and Tables

**Table 1:** Hydrogen-bond geometry (Å, °)

*D*—H⋯*A*	*D*—H	H⋯*A*	*D*⋯*A*	*D*—H⋯*A*
O1—H1⋯N1	0.82	1.93	2.649 (3)	145
N2—H2*A*⋯O2^i^	0.89 (1)	2.11 (2)	2.946 (3)	155 (3)
N2—H2*A*⋯O3	0.89 (1)	2.26 (3)	2.733 (3)	113 (2)

Symmetry code: (i) 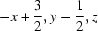.

**Fig. 1.**
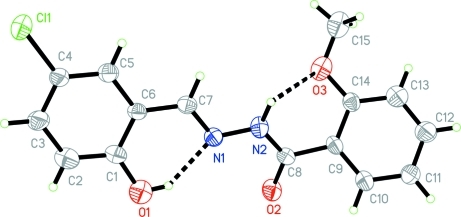
The molecular structure of the title compound with 30% probability ellipsoids. Intramolecular hydrogen bonds are drawn as dashed lines.

**Fig. 2.**
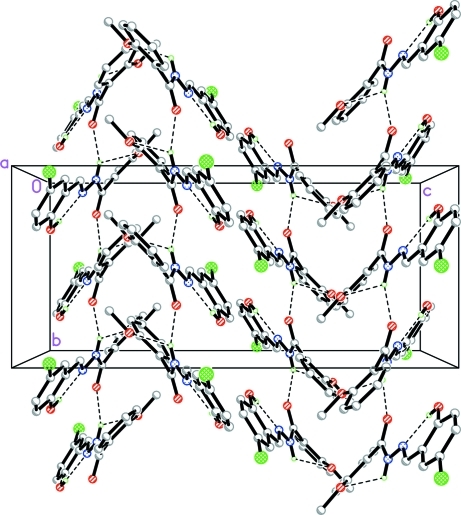
Molecular packing of the title compound with hydrogen bonds drawn as dashed lines.

## supplementary crystallographic information

### Comment

Schiff base compounds are a class of important materials used as pharmaceutical and medicinal fields (Sriram *et al.*,  2006; Karthikeyan *et al.*,  2006; Dao *et al.*,  2000). Schiff bases have also been used as versatile ligands in coordination chemistry (Ali *et al.*,  2008; Kargar *et al.*,  2009; Yeap *et al.*,  2009). Recently, the crystal structures of a large number of Schiff base compounds bearing the hydrazone groups have been reported (Fun *et al.*,  2009; Nadeem *et al.*,  2009; Eltayeb *et al.*,  2008). As a continuous work (Hao, 2009a,b,c,d; Hao, 2010), in this paper, the title Schiff base compound, Fig. 1, is reported.

In the title compound, the dihedral angle between the two benzene rings is 20.6 (3)°. All the bond lengths are within normal values (Allen *et al.*,  1987).

In the crystal structure, molecules are linked through intermolecular N—H···O hydrogen bonds (Table 1), forming chains along the b direction (Fig. 2).

### Experimental

5-Chloro-2-hydroxybenzaldehyde (0.1 mmol, 15.6 mg) and 2-methoxybenzohydrazide (0.1 mmol, 16.6 mg) were refluxed in a 30 ml methanol solution for 30 min to give a clear colorless solution. Colorless block-shaped single crystals of the compound were formed by slow evaporation of the solvent over several days at room temperature.

### Refinement

H2A was located from a difference Fourier map and refined isotropically, with the N—H distance restrained to 0.90 (1)Å, and with *U*_iso_ fixed at 0.08Å^2^. Other H atoms were constrained to ideal geometries, with d(C—H) = 0.93-0.96Å, d(O—H) = 0.82Å, and with *U*_iso_(H) = 1.2*U*_eq_(C) and 1.5*U*_eq_(O and C15).

### Crystal data

**Table:**

C_15_H_13_ClN_2_O_3_	*F*(000) = 1264
*M**_r_* = 304.72	*D*_x_ = 1.434 Mg m^−^^3^
Orthorhombic, *P**b**c**a*	Mo *K*α radiation, λ = 0.71073 Å
Hall symbol: -P 2ac 2ab	Cell parameters from 868 reflections
*a* = 15.392 (3) Å	θ = 2.4–24.5°
*b* = 9.110 (2) Å	µ = 0.28 mm^−^^1^
*c* = 20.128 (3) Å	*T* = 298 K
*V* = 2822.4 (9) Å^3^	Block, colorless
*Z* = 8	0.30 × 0.30 × 0.27 mm

### Data collection

**Table:**

Bruker SMART CCD area-detector diffractometer	3051 independent reflections
Radiation source: fine-focus sealed tube	1463 reflections with *I* > 2σ(*I*)
graphite	*R*_int_ = 0.067
ω scans	θ_max_ = 27.0°, θ_min_ = 2.4°
Absorption correction: multi-scan (*SADABS*; Sheldrick, 1996)	*h* = −19→9
*T*_min_ = 0.920, *T*_max_ = 0.928	*k* = −9→11
9958 measured reflections	*l* = −21→25

### Refinement

**Table:**

Refinement on *F*^2^	Primary atom site location: structure-invariant direct methods
Least-squares matrix: full	Secondary atom site location: difference Fourier map
*R*[*F*^2^ > 2σ(*F*^2^)] = 0.056	Hydrogen site location: inferred from neighbouring sites
*wR*(*F*^2^) = 0.150	H atoms treated by a mixture of independent and constrained refinement
*S* = 0.99	*w* = 1/[σ^2^(*F*_o_^2^) + (0.055*P*)^2^] where *P* = (*F*_o_^2^ + 2*F*_c_^2^)/3
3051 reflections	(Δ/σ)_max_ = 0.001
195 parameters	Δρ_max_ = 0.17 e Å^−^^3^
1 restraint	Δρ_min_ = −0.27 e Å^−^^3^

### Special details

**Table:**

Geometry. All esds (except the esd in the dihedral angle between two l.s. planes) are estimated using the full covariance matrix. The cell esds are taken into account individually in the estimation of esds in distances, angles and torsion angles; correlations between esds in cell parameters are only used when they are defined by crystal symmetry. An approximate (isotropic) treatment of cell esds is used for estimating esds involving l.s. planes.
Refinement. Refinement of F^2^ against ALL reflections. The weighted R-factor wR and goodness of fit S are based on F^2^, conventional R-factors R are based on F, with F set to zero for negative F^2^. The threshold expression of F^2^ > 2sigma(F^2^) is used only for calculating R-factors(gt) etc. and is not relevant to the choice of reflections for refinement. R-factors based on F^2^ are statistically about twice as large as those based on F, and R- factors based on ALL data will be even larger.

### Fractional atomic coordinates and isotropic or equivalent isotropic displacement parameters (Å^2^)

**Table:**

	*x*	*y*	*z*	*U*_iso_*/*U*_eq_	
Cl1	1.23358 (6)	1.00596 (10)	0.43870 (5)	0.0931 (4)	
N1	0.83185 (15)	1.0819 (2)	0.38838 (11)	0.0491 (6)	
N2	0.76629 (15)	1.0034 (2)	0.35851 (13)	0.0532 (7)	
O1	0.89608 (14)	1.2927 (2)	0.46463 (12)	0.0713 (7)	
H1	0.8575	1.2452	0.4466	0.107*	
O2	0.67478 (12)	1.1960 (2)	0.36350 (11)	0.0626 (6)	
O3	0.71657 (13)	0.8451 (2)	0.24964 (11)	0.0675 (6)	
C1	0.9728 (2)	1.2226 (3)	0.45691 (15)	0.0547 (8)	
C2	1.0460 (2)	1.2825 (3)	0.48644 (16)	0.0670 (9)	
H2	1.0410	1.3690	0.5107	0.080*	
C3	1.1255 (2)	1.2168 (4)	0.48063 (16)	0.0710 (10)	
H3	1.1741	1.2579	0.5007	0.085*	
C4	1.13245 (19)	1.0896 (4)	0.44478 (16)	0.0600 (8)	
C5	1.06147 (18)	1.0276 (3)	0.41535 (16)	0.0557 (8)	
H5	1.0676	0.9407	0.3916	0.067*	
C6	0.97993 (17)	1.0929 (3)	0.42047 (14)	0.0477 (7)	
C7	0.90686 (18)	1.0219 (3)	0.38947 (14)	0.0487 (7)	
H7	0.9144	0.9305	0.3698	0.058*	
C8	0.68942 (18)	1.0680 (3)	0.34816 (13)	0.0467 (7)	
C9	0.61993 (17)	0.9746 (3)	0.31858 (15)	0.0494 (7)	
C10	0.53588 (19)	1.0005 (3)	0.34012 (16)	0.0573 (8)	
H10	0.5259	1.0737	0.3714	0.069*	
C11	0.4666 (2)	0.9204 (4)	0.31633 (18)	0.0675 (9)	
H11	0.4108	0.9375	0.3322	0.081*	
C12	0.4811 (2)	0.8159 (4)	0.26919 (19)	0.0721 (10)	
H12	0.4346	0.7624	0.2524	0.086*	
C13	0.5630 (2)	0.7886 (3)	0.24629 (17)	0.0654 (9)	
H13	0.5716	0.7170	0.2140	0.078*	
C14	0.63371 (19)	0.8664 (3)	0.27054 (15)	0.0519 (8)	
C15	0.7318 (2)	0.7299 (4)	0.20290 (18)	0.0821 (11)	
H15A	0.7109	0.6388	0.2208	0.123*	
H15B	0.7930	0.7222	0.1943	0.123*	
H15C	0.7018	0.7513	0.1622	0.123*	
H2A	0.7748 (19)	0.9090 (14)	0.3487 (15)	0.080*	

### Atomic displacement parameters (Å^2^)

**Table:**

	*U*^11^	*U*^22^	*U*^33^	*U*^12^	*U*^13^	*U*^23^
Cl1	0.0545 (5)	0.1169 (8)	0.1077 (9)	−0.0004 (5)	−0.0070 (5)	0.0248 (6)
N1	0.0517 (14)	0.0446 (13)	0.0510 (16)	−0.0044 (11)	−0.0067 (12)	−0.0020 (12)
N2	0.0528 (14)	0.0403 (13)	0.0666 (18)	0.0001 (12)	−0.0152 (12)	−0.0101 (13)
O1	0.0792 (16)	0.0573 (13)	0.0775 (17)	0.0036 (11)	0.0003 (13)	−0.0150 (12)
O2	0.0662 (13)	0.0397 (11)	0.0821 (16)	0.0041 (9)	−0.0057 (11)	−0.0110 (11)
O3	0.0613 (13)	0.0702 (14)	0.0709 (15)	−0.0022 (10)	−0.0046 (12)	−0.0297 (12)
C1	0.070 (2)	0.0471 (18)	0.0470 (19)	−0.0062 (15)	0.0006 (16)	0.0012 (15)
C2	0.092 (3)	0.055 (2)	0.053 (2)	−0.0216 (18)	−0.0115 (18)	−0.0081 (17)
C3	0.068 (2)	0.084 (3)	0.062 (2)	−0.029 (2)	−0.0157 (18)	0.012 (2)
C4	0.0542 (18)	0.071 (2)	0.055 (2)	−0.0117 (16)	−0.0066 (16)	0.0146 (18)
C5	0.0583 (19)	0.0567 (18)	0.0521 (19)	−0.0077 (14)	−0.0017 (15)	0.0031 (16)
C6	0.0528 (17)	0.0460 (16)	0.0443 (18)	−0.0091 (13)	−0.0031 (14)	0.0041 (14)
C7	0.0556 (18)	0.0408 (16)	0.0497 (19)	−0.0043 (13)	0.0001 (14)	−0.0026 (14)
C8	0.0556 (18)	0.0415 (16)	0.0428 (18)	−0.0015 (13)	−0.0012 (14)	−0.0010 (14)
C9	0.0533 (17)	0.0428 (16)	0.0523 (19)	0.0023 (13)	−0.0116 (14)	0.0046 (15)
C10	0.060 (2)	0.0517 (18)	0.060 (2)	0.0040 (15)	−0.0089 (16)	0.0027 (15)
C11	0.0522 (19)	0.071 (2)	0.080 (3)	−0.0043 (16)	−0.0053 (18)	0.012 (2)
C12	0.063 (2)	0.070 (2)	0.082 (3)	−0.0143 (17)	−0.0148 (19)	0.006 (2)
C13	0.070 (2)	0.0587 (19)	0.068 (2)	−0.0113 (16)	−0.0126 (18)	−0.0106 (17)
C14	0.0526 (18)	0.0482 (17)	0.055 (2)	0.0008 (14)	−0.0085 (15)	−0.0001 (16)
C15	0.079 (2)	0.079 (2)	0.088 (3)	0.0057 (18)	−0.005 (2)	−0.033 (2)

### Geometric parameters (Å, °)

**Table:**

Cl1—C4	1.737 (3)	C5—H5	0.9300
N1—C7	1.278 (3)	C6—C7	1.439 (4)
N1—N2	1.375 (3)	C7—H7	0.9300
N2—C8	1.338 (3)	C8—C9	1.491 (4)
N2—H2A	0.892 (10)	C9—C10	1.385 (4)
O1—C1	1.351 (3)	C9—C14	1.397 (4)
O1—H1	0.8200	C10—C11	1.377 (4)
O2—C8	1.227 (3)	C10—H10	0.9300
O3—C14	1.357 (3)	C11—C12	1.363 (5)
O3—C15	1.429 (3)	C11—H11	0.9300
C1—C2	1.386 (4)	C12—C13	1.365 (4)
C1—C6	1.395 (4)	C12—H12	0.9300
C2—C3	1.367 (5)	C13—C14	1.387 (4)
C2—H2	0.9300	C13—H13	0.9300
C3—C4	1.369 (4)	C15—H15A	0.9600
C3—H3	0.9300	C15—H15B	0.9600
C4—C5	1.365 (4)	C15—H15C	0.9600
C5—C6	1.393 (4)		
			
C7—N1—N2	116.6 (2)	O2—C8—N2	122.8 (2)
C8—N2—N1	119.2 (2)	O2—C8—C9	120.8 (2)
C8—N2—H2A	121 (2)	N2—C8—C9	116.5 (2)
N1—N2—H2A	119 (2)	C10—C9—C14	118.6 (3)
C1—O1—H1	109.5	C10—C9—C8	116.6 (3)
C14—O3—C15	117.6 (2)	C14—C9—C8	124.8 (3)
O1—C1—C2	118.4 (3)	C11—C10—C9	121.6 (3)
O1—C1—C6	122.0 (3)	C11—C10—H10	119.2
C2—C1—C6	119.6 (3)	C9—C10—H10	119.2
C3—C2—C1	121.3 (3)	C12—C11—C10	119.1 (3)
C3—C2—H2	119.4	C12—C11—H11	120.5
C1—C2—H2	119.4	C10—C11—H11	120.5
C2—C3—C4	119.0 (3)	C11—C12—C13	120.9 (3)
C2—C3—H3	120.5	C11—C12—H12	119.6
C4—C3—H3	120.5	C13—C12—H12	119.6
C5—C4—C3	121.1 (3)	C12—C13—C14	120.8 (3)
C5—C4—Cl1	120.4 (3)	C12—C13—H13	119.6
C3—C4—Cl1	118.6 (3)	C14—C13—H13	119.6
C4—C5—C6	120.9 (3)	O3—C14—C13	123.7 (3)
C4—C5—H5	119.6	O3—C14—C9	117.3 (2)
C6—C5—H5	119.6	C13—C14—C9	119.0 (3)
C5—C6—C1	118.1 (3)	O3—C15—H15A	109.5
C5—C6—C7	118.7 (3)	O3—C15—H15B	109.5
C1—C6—C7	123.1 (3)	H15A—C15—H15B	109.5
N1—C7—C6	121.4 (3)	O3—C15—H15C	109.5
N1—C7—H7	119.3	H15A—C15—H15C	109.5
C6—C7—H7	119.3	H15B—C15—H15C	109.5

### Hydrogen-bond geometry (Å, °)

**Table:**

*D*—H···*A*	*D*—H	H···*A*	*D*···*A*	*D*—H···*A*
O1—H1···N1	0.82	1.93	2.649 (3)	145
N2—H2A···O2^i^	0.89 (1)	2.11 (2)	2.946 (3)	155 (3)
N2—H2A···O3	0.89 (1)	2.26 (3)	2.733 (3)	113 (2)

Symmetry codes: (i) −*x*+3/2, *y*−1/2, *z*.
